# Perspectives of South American physicians hosting foreign rotators in emergency medicine

**DOI:** 10.1186/s12245-014-0024-5

**Published:** 2014-08-02

**Authors:** Steve O’Donnell, David H Adler, Pholaphat Charles Inboriboon, Hermenegildo Alvarado, Raul Acosta, Daniel Godoy-Monzon

**Affiliations:** 1 Department of Neurology, University of Utah Health Sciences Center, Salt Lake City 84132, UT, USA; 2 Department of Emergency Medicine, University of Rochester School of Medicine and Dentistry, Rochester 14642, NY, USA; 3 Department of Emergency Medicine, School of Medicine, University of Missouri-Kansas City, Kansas City 64110, MO, USA; 4 Clínica San Pablo, Av. El Polo 789–Monterrico, Perú; 5 Department of Emergency Medicine, Cayetano Heredia University, Av. Honorio Delgado 350 Urb. Ingeniería, S.M.P Lima 31, Perú; 6 Trauma and Orthopedics, Hospital Italiano, Buenos Aires C1181ACH, Argentina

**Keywords:** Emergency medicine, Global health, International Health Elective, Residency education, International Emergency Medicine

## Abstract

**Background:**

Emergency Medicine (EM) is increasingly becoming an international field. The number of fellowships in International EM in the USA is growing along with opportunities to complete international health electives (IHEs) during residency training. The impact on host institutions, however, has not been adequately investigated. The objective of this study is to assess the experience of several South American hospitals hosting foreign EM residents completing IHEs.

**Methods:**

Anonymous, semi-structured one-on-one interviews were conducted with physicians working in Emergency Departments in three hospitals in Lima, Peru and one hospital in Buenos Aires, Argentina. All participants reported previously working with EM foreign rotators. Interviews were analyzed qualitatively and coded for common themes.

**Results:**

Three department chairs, six residents, and 15 attending physicians were interviewed (total = 24). After qualitative analysis of interviews, two broad theme categories emerged: Benefits and Challenges. Most commonly reported benefits were *knowledge sharing about emergency medical systems* (78%), *medical knowledge transfer* (58%), and *long-term relationship formation* (42%). Top challenges included *rotator Spanish language proficiency* (70%) *lack of reciprocity* (58%), and *level of training and rotation length* (25%). Spanish proficiency related directly to how involved rotators became in patient care (e.g., taking a history, participating in rounds) but was not completely prohibitive, as a majority of physicians interviewed felt comfortable speaking in English. *Lack of reciprocity* refers to the difficulty of sending host physicians abroad as well as failed attempts at building long-lasting relationships with foreign institutions. Lastly, 25% preferred rotators to stay for at least 1 month and rotate in the last year of EM residency. This latter preference increased knowledge transfer from rotator to host.

**Conclusions:**

Our research identified benefits and challenges of IHEs in Emergency Medicine from the perspective of physician hosts in several hospitals in South America. Our results suggest that IHEs function best when EM residents rotate later in residency training and when relationships are maintained and deepened among those involved including host physicians, rotators, and institutions. This leads to future rotators, project collaboration, research, and publications which not only benefit individuals involved but also the wider field of Emergency Medicine.

## Background

Every year, physicians enrolled in residency training programs from the USA fan out across the globe to complete International Health Electives (IHEs) in a myriad of institutions that include urban academic medical centers, rural hospitals, and smaller clinics. The manner in which these opportunities are found varies. Some residents independently contact international institutions, others utilize extant electives offered by their home residency programs, and some choose to work with non-governmental organizations operating in a particular area of the world.

Emergency Medicine (EM), in particular, is becoming an increasingly international field. In the USA, 24% of EM residency training programs offer fellowships in global health and, according to a 2011 study, 71% have global health partnerships and International Health Electives [1],[2].

Effects of IHEs on the education and possible career trajectories of medical students and internal medicine resident physicians have been reported. Previous research revealed IHEs increased interest in primary care among medical students, made students more likely to select a residency training program with international opportunities, and created positive attitudinal changes towards public health and cross-cultural communication [3].

A study of resident physicians from a number of different specialties at the Mayo Clinic found educational and personal benefits of IHEs. Educationally, residents encountered a wider variety of pathology than at home institutions, learned to work with limited resources, and saw patients with a wide scope of pathologies and illness that often were not common in the USA. Personally, resident physicians enjoyed experiencing new people and cultures, appreciated the gratitude of the patients they saw, and were exposed to a different level of poverty and suffering since most experiences took place in the developing world [4]. Such research, however, has focused only on the effects on the resident physicians, or rotator, and not the physicians who play host. In addition, the studies above have not been specific to the field of Emergency Medicine.

What do institutions and physicians hosting residents completing an International Health Elective gain from the experience? What are the benefits, *to host physicians particularly*, of engaging in such activities? What are the challenges? To our knowledge, no study has sought to answer these questions.

## Methods

### Study design

This qualitative research project was created with the objective of evaluating the experience of physicians working in Emergency Departments that host foreign rotating Emergency Medicine residents completing International Health Electives. Our hypothesis was that institutions and physicians electing to act as hosts must experience benefits from participating in IHEs since the number of IHEs continues to grow. We also imagined that there were inherent problems and barriers. Thus, our research question became: What are the specific benefits and challenges to physician hosts participating in International Health Electives in Emergency Medicine?

An initial literature search was conducted on PubMed looking for previous research on the topic. Search terms included ‘International Educational Exchanges’ , ‘International Medical Education’ , ‘International Emergency Medicine’ , ‘International Health Elective’ , ‘Global Health Elective’ , ‘Global Health’ , AND Emergency Medicine.’ The search yielded no articles that focused particularly on host institution and physician experiences with IHEs in Emergency Medicine.

The next question became how best to acquire qualitative data for the study. An e-mail survey was contemplated but the authors were concerned over low response rates and lack of in-depth answers. Using a Qualitative Research Methods reference, the authors decided the methodology that would yield the most in-depth qualitative data would be face-to-face interviews with physician hosts using a survey that would act as a data guide during stakeholder interviews [5]. A qualitative survey was designed to evaluate the experience of physician hosts involved in EM IHEs and was translated into Spanish by a professional translator (Tables 1 and 2).

**Table 1:** Guiding questions, in English, for semi-structured interviews

	**Questions for Latin American staff**
1	Describe your experience working with physicians from the U.S.
2	What are the benefits of having U.S. physicians work in your hospital? Detriments?
3	Do you plan to train in another country in the future? If so, please describe these plans.
4	How does your residency program support international educational opportunities for residents?
5	Does your program host residents/physicians from foreign countries? If so, please describe these programs.
6	Describe what international residents do at your institution. What are their responsibilities?
7	What are the benefits from hosting international residents?
8	What are the barriers/difficulties?
9	What do you believe to be the importance of international educational experiences in EM residency training today?
10	What are your plans for future international educational exchanges?
11	With whom else should I talk to about these issues?

**Table 2:** Guiding questions, in Spanish, for semi-structured interviews

	**Preguntas para el Personal Latino Americano**
1	Describa su experiencia trabajando con los médicos de los Estados Unidos.
2	¿Cuáles son los beneficios de tener médicos de los Estados Unidos que trabajan en su hospital? ¿Desventajas?
3	¿Tiene usted planes para capacitarse en otro país en el futuro? Si este es el caso, por favor describa estos planes.
4	¿Cómo apoya su programa de residencia las oportunidades educativas internacionales para los residentes?
5	¿Acepta su programa residentes/médicos de países extranjeros? Si este es el caso, por favor describa estos programas.
6	Describa lo que los residentes internacionales hacen en su institución. ¿Cuáles son sus responsabilidades?
7	¿Cuáles son las experiencias beneficiosas de aceptar residentes internacionales
8	¿Cuáles son las barreras/dificultades?
9	¿Qué cree usted es la importancia de experiencias educativas internacionales en la capacitación en la residencia para Médicos de Emergencia hoy día?
10	¿Cuáles son sus planes para intercambios educativos internacionales en el futuro?
11	¿Con cuál otra persona tendría que hablar yo sobre estos asuntos?

South American physicians known to be active in the International Section of the American College of Emergency Physicians (ACEP) were contacted by e-mail and asked if they would be willing to collaborate in such an investigation. A physician in Peru and one in Argentina agreed to host the lead author and facilitate one-on-one physician interviews. The lead author traveled to South America to conduct stakeholder interviews from January to April of 2012.

### Hospitals

Interviews were conducted at Cayetano Heredia, Dos De Mayo, and Maria Auxiliadora hospitals in Lima, Peru and Hospital Italiano in Buenos Aires, Argentina. The majority of interviews occurred at two sites: Cayetano Heredia and Hospital Italiano.

Cayetano Heredia is a leading academic teaching hospital in Peru. It has an associated medical school and residency training programs in all major medical specialties including one in Emergency Medicine. It also hosts the internationally renowned Gorgas in Tropical Medicine Course that attracts physicians from around the world. Cayetano’s EM residency program trains one to two residents per year. Emergency Medicine is a relatively new specialty in Peru, in existence for only 10 to 15 years. Given this fact, most physicians caring for patients in the Emergency Department (ED) have training in either internal medicine or surgery. Because of Cayetano’s robust international connections, physicians in the ED frequently have worked with foreign rotators that include Emergency Medicine residents from the USA. In addition, Cayetano hosts medical students, resident physicians, and physicians from North America and Europe in specialties that range from Emergency Medicine, Internal Medicine, Infectious Disease, and Pediatrics among others.

Hospital Italiano is an academic private hospital in Buenos Aires, Argentina that mostly serves patients with some form of private health insurance who tend to be employed and among the more affluent members of the society. It has a medical school and residency training programs in all medical specialties, including one in Emergency Medicine, and is associated with the Universidad Nacional de Buenos Aires. The Emergency Department has hosted EM resident physicians from the USA and Canada. It also hosts physicians-in-training from other South American countries and Western Europe in Emergence Medicine, Pediatrics, and Internal Medicine. The trauma surgery service, which works closely with the Emergency Department, hosts Orthopedic Surgery residents from around the world including Western Europe and North America. In addition, faculty and residents in all specialties are encouraged to travel internationally for conferences, electives, and fellowship opportunities.

Dos de Mayo and Maria Auxiliadora are public hospitals in Lima, Peru.

### Interviews

Residents, attending physicians, and department chairs in each Emergency Department visited were asked if they would be willing to be interviewed. An information letter about the study in English and Spanish was provided and a time for the interview agreed upon. Two potential interviewees could not participate because of scheduling conflicts. Otherwise, all those approached to participate did so.

One-on-one interviews were performed with an interview guide (Tables 1 and 2). Follow-up questions were posed on individual topics that arose during the conversations. The majority of interviews were in English as most physicians felt comfortable speaking the language. A minority of interviews were conducted in Spanish. Notes taken during each interview were transcribed and saved electronically on the same day.

Then the ‘snowball sampling method’ - also known as the chain referral sampling - was utilized to identify study participants. In this method, initial contacts made ‘use their social networks to refer the researcher to other people who could potentially participate in or contribute to the study’ [5]. Two of the co-authors, Dr. Hermenegildo Alvarado and Dr. Godoy-Monzon, who work in the Emergency Departments in Peru and Argentina, respectively, initially referred the lead author to several physicians to interview. Each study participant was asked if he or she knew of other physicians that might want to take part and this led to additional interviews.

At the investigation’s conclusion, all interview transcripts were reviewed, analyzed qualitatively, and coded for common themes according to qualitative research principles [5]. The qualitative survey that acted as an interview guide ensured discussion of specific topics (Tables 1 and 2). The authors had no pre-conceived themes in mind and specifically made survey questions broad and open-ended. For example, question 8 from Table 1 asks, what are the barriers/difficulties of hosting international residents? A repeated answer was that rotators with limited Spanish fluency faced increased challenges engaging in direct patient care. The authors reviewed all interview transcripts and counted the number of participants who specifically mentioned language as a challenge and this topic then became a qualitative theme reported in the results section below.

The study received Institutional Review Board approval from the University of Rochester IRB.

## Results

**Figure 1:**
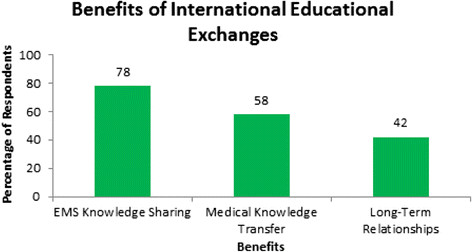
Percentages of South American physician interviewees mentioning specific benefits while hosting foreign rotators in ED.

**Figure 2:**
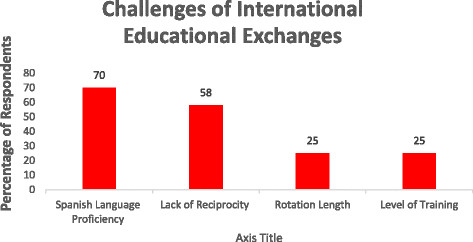
Percentages of South American physician interviewees mentioning specific challenges while hosting foreign rotators in ED.

Three department chairs, 6 residents, and 15 attending physicians were interviewed. All had worked with Emergency Medicine residents from North America completing an International Health Elective. Two broad theme categories, benefits and challenges, emerged to describe hosts’ experiences of international educational exchanges. Quotations from themed categories are listed in Tables 3 and 4 while Figures 1 and 2 are graphs of the percentage of respondents who mentioned a particular theme.

**Table 3:** Benefits to hosts participating in international educational exchanges

**Benefit**	**Quotations from interviews**
Knowledge sharing about EM systems and practice	‘Learning about how another system works in different countries is great and it makes you think about your own system, its administration, and what advantages new technology can bring’.
Medical knowledge transfer	‘A visiting resident taught us how to start central lines with ultrasound guidance. Now several of us use ultrasound whenever possible to start our central lines’.
Long-term relationship formation	‘My department maintains contact with the physicians who have worked here from around the world. Over time we have built a network that produces research projects and future exchanges’.

‘Benefits’ theme quotations from in-person interviews with South American physicians working in Emergency Departments hosting Emergency Medicine residents and other foreign rotators (total interviews = 24).

**Table 4:** Challenges to hosts participating in international educational exchanges

**Challenge**	**Quotations from interviews**
Language proficiency	‘The more you can speak Spanish, the more you can participate with interviewing patients and the medical team’.
Lack of reciprocity	‘It would be invaluable for our residents to visit the U.S. to understand where we are, where we could be, where we have to go, and how we have to improve but to do so requires finding a residency program to work with and the necessary finances’.
Length of stay	‘They should stay more than a few weeks to better know the hospital, the people, the culture, the city’.
Level of training	‘Rotators should come later in residency. When you travel abroad you should not seek to learn how to practice medicine, but instead learn how medicine is practiced in another country’.

‘Challenges’ theme quotations from in-person interviews with South American physicians working in Emergency Departments hosting Emergency Medicine residents and other foreign rotators (total interviews = 24).

### Benefits of international educational exchanges to hosts

#### Knowledge sharing about emergency medical systems and practice

This was the most common perceived benefit, identified by 78% of those interviewed. Hosts enjoy comparing the medical systems of host and rotator countries, particularly variances in the delivery of pre-hospital care in response to an emergency, methods of triage when a patient arrives to the ED, and the utilization and implementation of guidelines for different clinical situations. Distinct patient populations and antibiotic availability in South America and in Peru especially, compared to the ‘developed world’ , necessitated different clinical approaches. Often a combination of guidelines from the USA, Europe, and World Health Organization was used to develop institutional practice. Hosts liked sharing with rotators how their hospitals’ clinical practices evolved and were curious to compare these clinical practices with those in the USA and Europe.

#### Medical knowledge transfer

Fifty-eight percent reported medical knowledge transfer in the form of discussions, lectures, or skills demonstrations given by visiting residents as a benefit. For example, at one hospital an EM resident from the USA taught the placement of central lines with ultrasound guidance. After the resident departed the practice was continued and maintained.

The faculty at another hospital had collaborated with an EM physician from the University of Arizona to help create an online EM curriculum in Spanish geared towards residents and physicians wishing to engage in continuing medical education. The website, www.reeme.arizona.edu, is particularly popular in Latin America where access to continuing medical education is not as abundant as in the USA or Europe.

Overall, interviewees said education was bidirectional, flowing from rotator to host and host to rotator. Hosts repeatedly relayed that they enjoyed teaching rotators about infectious disease processes uncommon in the developed world as well as procedures and clinical practices utilized in the host hospital.

#### Long-term relationship formation

Long-term relationship formation can be viewed both as a benefit and a challenge: a benefit when a relationship was solidified and maintained and a challenge when it was not. Discussions in interviews often touched upon the development of relationships.

Forty-two percent of the participants mentioned that a successful international educational exchange occurred when a long-term relationship was formed, which could be with an individual, institution, or both. When asked why this was a positive, participants responded that it could lead to other rotators in the future, project creation and collaboration, and research.

Often, foreigners brought expertise and experience in research while the host provided a different clinical setting and patient population. For example, one site was conducting a clinical trial of an anti-coagulant drug that had completed phased clinical trials in the USA, and early results showed different rates of drug efficacy in a South American cohort.

### Challenges of international educational exchanges to hosts

#### Rotator Spanish language proficiency

This was the most common challenge, mentioned by 70% of interviewees. The issue of language related specifically to the realm of direct patient care. Spanish fluency is certainly not a requirement for completing an IHE in South America, and a majority of physicians interviewed spoke English with comfort, especially since most must pass an English language proficiency exam in order to graduate from medical school. But when rotators want to participate in the care of patients, as they understandably do during an elective, language can become a barrier. If a rotator has little to no Spanish proficiency, it becomes necessary to pair the rotator with a bilingual physician or to provide a translator. It is much easier to have rotators see patients and integrate them into the medical team if they possess intermediate to advanced mastery of Spanish. It was intrinsically understood by most South American physicians in the survey that part of the reason that some rotators came to South America was to work on language skills. One ED chair explicitly encouraged concomitant clinical rotations and language classes during electives.

#### Lack of reciprocity

Fifty-eight percent of host physicians mentioned something that was placed into this themed category. For example, one department chair wished his EM residents were able to do a rotation in the USA. This, however, was prohibitively difficult due to the high cost and bureaucratic barriers of entrance to the USA as well as reluctance on the part of some US hospitals to host foreign physicians.

As mentioned in the benefits section above, the hosts desired the creation of long-lasting relationships. Many interviewees, however, stated that some rotators came and were never heard from again and contacts with outside institutions broke down. When these things occurred, it was a source of frustration and viewed as a loss of investment of time and effort.

#### Level of training and rotation length

Twenty-five percent said they wanted a rotator to stay for at least a month. This allowed time for the hosts and rotator to adapt to one another and for the rotator to learn about, and be integrated into, the host’s medical system.

In addition, the same percentage of hosts preferred when residents came in the later stages of training or to have an attending accompanying residents. This relieved the host from having to teach basic skills to residents and facilitated increased knowledge transfer from rotator to host. One institution asked every resident to prepare a talk on a topic of their choice to present during grand rounds or similar didactic conference.

## Discussion

We believe this qualitative research study is one of the first to report on benefits and challenges experienced by physicians who host Emergency Medicine residents completing International Health Electives. From 24 interviews with physician hosts, themed benefits and challenges of IHEs emerged. Benefits included knowledge sharing about Emergency Medical Systems and practice, bidirectional knowledge acquisition and education, and long-term relationship formation. Reported challenges included language proficiency, lack of reciprocity, level of training of the resident, and length of stay by the resident. Four Emergency Departments in South America, specifically in Peru and Argentina, were the sites for this investigation.

Collectively, the benefits listed above may aid in the better delivery of emergency medical care throughout the world in different resource settings. A systematic review of existing research is the hallmark of advancing medical practice. It is the basis upon which clinical guidelines are created. But as Arnold and colleagues point out, the majority of studies underpinning reviews are performed in countries with sophisticated health care systems, ‘thus not necessarily reflecting the burden of disease or resources available in developing economies’ [6]. Our research suggests that participants in IHEs, both hosts and rotators, gain broader knowledge of the diversity of Emergency Medical Systems. Long-term relationships also lead to local research projects that have more relevance to host populations. Hence, IHEs represent one important mechanism of adapting existing EM guidelines and protocols to varying local realities throughout the world and for building a broader evidence base upon which general, widely implementable emergency medical care principles can be created.

We believe that the identified challenges are not prohibitive to the continued expansion of these experiences. The Council of Emergency Medicine Resident Directors (CORD), during their 2011 Academic Assembly, developed consensus recommendations to enhance the global health education of Emergency Medicine residents. They recommended standardizing global health application materials, standardizing resident evaluations during global health rotations, creating a global health site database, and incorporating a method to evaluate global health sites [2]. We support standardization, evaluation, and transparency, and believe they will only improve IHEs in the future.

Implicit in CORD’s recommendations, and necessary for success in our opinion, is the continual input and feedback from those who host EM trainees. A thorough understanding of the IHE experience - from the perspectives of both host and rotator - will ultimately lead to the design of electives that yield the highest educational benefit.

Additionally, we would recommend that US EM professional organizations continue to offer, and increase when possible, scholarships and grants for foreign physicians to rotate in US hospitals.

### Limitations

This qualitative research project investigates physician host experiences of IHEs offered to foreign EM residents in four hospitals in Peru and Argentina. It is uncertain how generalizable and applicable these results are to other places in the world where IHEs occur. In addition, only large, urban hospitals were visited and thus the results might not be germane to smaller hospitals or more rural health facilities.

As mentioned in the Methods section, participants in the study were identified utilizing the snowball method whereby initial in-country contacts refer the investigator to potential additional contacts. Snowball sampling will always have selection bias because of purposive sampling. This is mitigated by the wide variety of subjects identified and by having a mixed team of researchers from several countries.

The number of interviews obtained was constrained by the finite amount of time the lead author had at each site and the availability of participants in that time frame. Therefore, the data is susceptible to some convenience sampling. However, in three out of four sites, an interview with the Emergency Department Chair was obtained which provided consistent viewpoints from those in leadership positions. In addition, out of all interviews attempted, only two could not be performed due to scheduling conflicts.

Finally, this qualitative research study does not comprehensively assess EM IHEs. As identified by the 2011 CORD Academic Assembly, a helpful step would be to collect and analyze evaluation data from residents who completed global health rotations throughout the world. Furthermore, a future investigation that tracked EM residents after they completed global health rotations to understand how these experiences impacted future career trajectories would be helpful to better comprehend the impact of IHEs.

## Conclusions

Our research identified benefits and challenges of international educational exchanges in Emergency Medicine from the perspective of physician hosts in several hospitals in South America. Our results suggest that International Health Electives function best when relationships are maintained and deepened among those involved including host physicians, rotators, and institutions. This leads to the expression of clear expectations from all parties of what the international health elective should entail. Relationship maintenance creates opportunities for more rotators in the future, maximal educational benefits for rotator and host, and the possibility to collaborate on new projects and research. Thus, IHEs have the potential to expand the EM knowledge base, to adapt existing EM guidelines and protocols to local realities, and to identify broadly applicable principles of emergency medical care in multiple resource settings.

Interest among US EM trainees in participating in international electives is on the rise. Almost three-quarters of US EM residencies report having opportunities in global health and about one-quarter offer a fellowship specifically in the field [1],[2]. As participation in global health by US trainees continues to increase, future work should strive to ensure the optimization of global health opportunities. The Council of Emergency Medicine Residency Directors’ recommendations would help standardize the global health elective process for residents. Additional feedback from residents when they return from abroad as well as tracking their future career trajectories would lead to better understanding of the impact of global health experiences. Finally, it is vital to consistently elicit input and perspective from those who graciously agree to serve as host.

## Competing interests

The authors declare that they have no competing interests.

## Authors’ contributions

SO traveled to the hospitals mentioned in the study and conducted one-on-one stakeholder interviews. SO, DA, and PI collaborated on study design, surveys used for interviews, and qualitative data analysis. HA, RA, and DM helped identify the sites visited in the study and provided feedback and advice for study implementation. All authors read and approved the final manuscript.
